# RETRACTED: Zulfiqar et al. Obesity and Frailty Syndrome in the Elderly: Prospective Study in Primary Care. *Medicines* 2022, *9*, 38

**DOI:** 10.3390/medicines11080022

**Published:** 2024-12-18

**Authors:** Abrar-Ahmad Zulfiqar, Perla Habchi, Ibrahima Amadou Dembele

**Affiliations:** 1 Service de Médecine Interne, Diabète et Maladies Métaboliques de la Clinique Médicale B, Hôpitaux Universitaires de Strasbourg et Equipe EA 3072 “Mitochondrie, Stress Oxydant et Protection Musculaire”, Faculté de Médecine-Université de Strasbourg, 67000 Strasbourg, France; ibrahimaadembele@gmail.com; 2 Anesthesiology Consultant, Aman Hospital, F Ring Rd., Zone 47, Building 412, Doha P.O. Box 8199, Qatar; p.habchi@amanhospital.org

The journal retracts the article titled “Obesity and Frailty Syndrome in the Elderly: Prospective Study in Primary Care” [1], cited above.

Following publication, concerns were brought to the attention of the Editorial Office regarding the extent to which this article [1] adheres to national regulations governing research involving human participants.

Adhering to our complaints procedure, the Editorial Office and Editorial Board conducted an investigation that concluded that this study falls under a prospective observational study classification, which requires validation and approval from the national ethics committee (CPP) before patient inclusion according to the guidance of the French laws for clinical research. Taking into consideration that this study is missing mandatory ethical validation from the CPP, the Editorial Board concluded that this study does not adhere to the national regulations governing research involving human participants. As a result, the Editorial Board have concluded that this study did not receive the required level of ethical oversight and have decided to retract this publication [1], as per MDPI’s retraction policy (https://www.mdpi.com/ethics#_bookmark30).

This retraction was approved by the Editor-in Chief of the *Medicines* journal.

The authors did not agree to this retraction.

## References

[1] Zulfiqar A.-A., Habchi P., Dembele I.A. (2022). RETRACTED: Obesity and Frailty Syndrome in the Elderly: Prospective Study in Primary Care. Medicines.

